# An Efficient Distributed Coverage Hole Detection Protocol for Wireless Sensor Networks

**DOI:** 10.3390/s16030386

**Published:** 2016-03-17

**Authors:** Prasan Kumar Sahoo, Ming-Jer Chiang, Shih-Lin Wu

**Affiliations:** 1 Department of Computer Science and Information Engineering, Chang Gung University, Kwei-Shan 33302, Taiwan; pksahoo@mail.cgu.edu.tw; 2 Department of Electrical Engineering, Chang Gung University, Kwei-Shan 33302, Taiwan; d9621006@stmail.cgu.edu.tw; 3 Center for Biomedical Engineering, College of Engineering, Chang Gung University, Kwei-Shan 33302, Taiwan

**Keywords:** wireless sensor networks, coverage, hole detection

## Abstract

In wireless sensor networks (WSNs), certain areas of the monitoring region may have coverage holes and serious coverage overlapping due to the random deployment of sensors. The failure of electronic components, software bugs and destructive agents could lead to the random death of the nodes. Sensors may be dead due to exhaustion of battery power, which may cause the network to be uncovered and disconnected. Based on the deployment nature of the nodes in remote or hostile environments, such as a battlefield or desert, it is impossible to recharge or replace the battery. However, the data gathered by the sensors are highly essential for the analysis, and therefore, the collaborative detection of coverage holes has strategic importance in WSNs. In this paper, distributed coverage hole detection algorithms are designed, where nodes can collaborate to detect the coverage holes autonomously. The performance evaluation of our protocols suggests that our protocols outperform in terms of hole detection time, limited power consumption and control packet overhead to detect holes as compared to other similar protocols.

## 1. Introduction

With the advance of microelectromechanical systems (MEMS) technology, wireless sensor networks (WSNs) [1] play an important role in transportation, infrastructures and forest monitoring for animals or fire [2,3], with its inexpensive, small size and multi-functional abilities. Normally, a wireless sensor network is composed of a large number of sensors deployed over the monitoring region regularly or randomly, and the deployed sensors are self-organized [4] to form the network. Since a random deployment is usually used in WSNs, the study of coverage issues is very important, which should be studied deeply in addition to the localization, target tracking and time synchronization in WSNs. However, a sensor node is severely constrained by the resources, such as limited memory, battery power, computation and communication capabilities. The energy consumption of each sensor is a function of its own sensing and communication range, and designing algorithms for deployment, localization, duty cycle, coverage and other issues is most important in WSNs.

The sensors are deployed randomly over a monitoring region with a high degree of density of nodes. Due to the random deployment strategy, certain areas of the monitoring region may have coverage holes [5,6] or serious coverage overlapping, which significantly degrade the network performance. Besides, the failure of electronic components, software bugs and destructive agents could lead to the random death of the nodes, and also, nodes may die due to the exhaustion of battery power, which may cause the network to be uncovered and disconnected. Since sensors are deployed in remote or hostile environments, such as a battlefield or desert, it is impossible to recharge or replace the battery. However, the data gathered by the sensors are highly critical and may be of scientific or strategic importance. Hence, the coverage provided in the sensor networks is a critical criterion of their effectiveness, and its maintenance is highly essential to form a robust network. The study of such coverage issues means summing of the sensing and communication range, which should be ideal in the monitoring area to provide good QoS for different applications.

Sensing coverage is a fundamental problem in WSNs and has been well studied over the past few years. However, most of the previous works address only one kind of redundancy, *i.e*., sensing or communication alone. The authors in [7] address how to combine coverage and connectivity maintenance in a single activity scheduling. In this work, it is proven that the communication range is at least twice the sensing range, which is the sufficient condition to ensure that a full coverage of a convex area implies connectivity among active nodes. In WSNs, nodes are deployed randomly, and it is hard or impossible to guarantee complete coverage of the monitored region, even if the node density is very high. Based on the deployment nature of the wireless sensor networks, the authors in [8] consider the communication range to be twice the sensing range, which is the sufficient condition and tight lower bound to ensure that the complete coverage preservation implies connectivity among active nodes, if the original network topology is connected.

In WSNs, due to the random deployment of the nodes, some areas cannot be covered [9,10,11] or have no connectivity. If a coverage region is sensed by at least *k* nodes, this sensing method is called *k*-covered, and the maintenance of a *k*-covered region is discussed in [12,13,14]. The coverage probability plays a fundamental role in coverage hole detection and other applications of WSNs, because of existing errors in the sensing and communication range [15,16]. Holes are hardly avoided in WSNs due to the various geographical environments of the monitoring region, such as the presence of ponds, obstacles or even due to physical destruction of the nodes. Ignoring the detection of holes affects the efficiency of geographic routing, data congestion and the excessive energy consumption of the hole boundary nodes. Additionally, for information flow, the hole could also affect the overall capacity of the network. Thus, the identification of the holes in sensor networks is of primary interest, because their presence often has a physical correspondence and may also map to one of the special events that are being monitored by the sensor networks.

Depending on the application environments and the level of information constraints, algorithms for identifying various coverage holes in the sensor networks can be generally classified into three categories: the computational geometry approach, the statistical approach and the topological method. In this paper, energy-efficient coverage hole detection algorithms are developed for wireless sensor networks, which can detect the presence of coverage holes distributively. The proposed hole detection methods can be achieved by considering the theoretical analysis of the point of intersection of the sensing discs of the sensors. First, the set of nodes that encloses a coverage hole is identified. This is done by taking location information of the nodes, their sensing coverage overlap and the circumference of the non-overlapping region of the sensors. Then, the coverage hole is detected by collaborating with the one-hop neighbors of the nodes that enclose the hole.

The rest of this paper is organized as follows. Related work is presented in Section 2. Section 3 describes the system model for detecting the holes in the WSNs. The proposed hole detection protocol is given in Section 4. The performance evaluation of the proposed protocol is done in Section 5, and concluding remarks are made in Section 6.

## 2. Related Work

Hole detection is one of the important research issues in WSNs [10], which can be classified as coverage holes, routing holes, jamming holes, sink/black holes and worm holes. Due to the random deployment of the sensors, some part of the monitoring region is not covered by the sensing disc of a sensor, which is called a coverage hole. The studies on coverage can be divided into two categories. The first category comprises probabilistic approaches for calculating the required node density for ensuring appropriate coverage [17], though these studies do not prove the hole detection solutions. The second category utilizes computational geometry approaches to discover the coverage holes. In this category, the detected holes problem, which is also called the connected coverage boundary detection problem, is addressed in [18]. Such a connected coverage boundary detection problem is classified into polygon-based and perimeter-based approaches. In the polygon-based approach, a Voronoi diagram is used for the coverage boundary detection. Briefly speaking, the VP of a node set *V* is the partition of the Euclidean space into polygons, called Voronoi polygons (VPs). According to the closeness property, if some portion of the VP is not covered by the nodes inside the VP, it will not be covered by any other node, which implies a coverage hole. However, it has been shown that the VPs of the boundary nodes cannot be locally computed, and the VP-based approach is not a real localized solution. The paper in [18] proposes a localized Voronoi polygon (LVP) to find the holes locally.

The first localized boundary node detection algorithm for the perimeter-based approach is proposed in [19], which is based on the information about the coverage of the perimeter of each node’s sensing disk. According to the perimeter-based approach, if a node *i* is the boundary node that exists at one point *j* on the circle, then point *j* is not covered by any one of the node *i*’s neighborhood sensors. The paper [20] proposes to check the intersection point by the node *i* and *i*’s neighborhood sensors not covered by other sensors. In [21], a coverage configuration protocol (CCP) based on the perimeter-based approach is presented to calculate the degree of the intersection points and to use this degree of intersection to maintain the *k*-coverage. In this paper, we define these special intersection points as hole points. However, the perimeter-based approach has two flaws. First, each node needs to check the positions and status of all of its neighbors, which is inefficient when the sensor nodes are densely deployed, and every time when a node dies, all of its neighbors need to check the coverage of their perimeters to update the existing coverage. Secondly, this approach is not efficient for using this information only to check the boundary nodes. In [22], the authors propose algorithms to construct two sets *P* and *Q* using the perimeter-based approach, where *P* is the end point set and *Q* is the edge point set, and the hole exists if two consecutive end points are found in the counterclockwise direction. However, it has two problems. The first problem is it needs more memory to store this information, and the second problem is it cannot solve the flaws of the perimeter-based approach. In [23], the authors propose a vector method to use the perimeter-based approach to group the hole points and to recover the coverage holes. Though they use the hole point information to recover the coverage holes, they do not consider the redundant sensors to recover them.

The formation of holes in the target field is quite common and is unavoidable due to the nature of the WSN and random deployment. This is very important to ensure that the target field is completely and continuously covered. In [24], the authors propose an approach called the hybrid hole detection and healing (HHDH) protocol that detects and heals the coverage holes effectively with minimum sensor movements. This protocol can recover the coverage hole formed due to the random deployment of the nodes. In [25], the authors use the knowledge of the location of each node for detecting coverage holes in randomly-deployed wireless sensor networks. In [26], the authors present a novel method for coverage hole detection considering the residual energy of the nodes in randomly-deployed wireless sensor networks. In this paper, by calculating the life expectancy of working nodes through the residual energy, the authors make a trade-off between the network repair cost and energy waste in which the working nodes with a short lifetime are screened out according to a proper ratio. As one of the best health indicators of the sensor network, the coverage holes directly decide the quality of the sensor network. In [27], the authors firstly propose an active contour model-based coverage hole detection algorithm for the sensor network, which can accurately evaluate both the number and the size of the holes.

The hybrid deployment of WSNs with static and mobile nodes in the monitoring area is an important issue to cover a maximum sensing area with a limited number of nodes. Furthermore, mobile sensor nodes can relocate themselves to improve the coverage area in the network. In [28], the authors propose a method that reduces the complexity of the relocation of the initial deployment and coverage hole healing of mobile sensor nodes in the hybrid WSNs. Their method finds the ways to get the shortest distance movements for the mobile nodes in WSNs. An adaptive threshold distance is used to eliminate some mobile nodes, which are already occupied or situated within the threshold distance from the optimal new positions. In [29], the authors develop distributed algorithms to detect and localize the coverage holes in sensor networks. This paper uses algebraic topological methods to define a coverage hole and develops algorithms to detect a hole. In [30], the authors design a stochastic learning weak estimation-based scheme, namely mobility prediction inside a coverage hole. The main objective of this scheme is that it could be able to correctly predict the mobility pattern of a target inside a coverage hole with low computational overhead.

In [31], the authors propose an intelligent strategy called the improved hybrid particle swarm algorithm to repair the coverage holes. Taking a hybrid sensor network, the authors consider the displacement of mobile sensors, energy consumption and energy balancing together. Their proposed protocol can schedule the redundant mobile sensors effectively to repair the coverage holes by moving them to appropriate locations. Besides, they improve the event detection rate to calculate the event detection lost or the detection error and coverage holes that seriously damage the quality of monitoring in WSNs. In [32], the authors design a localized coverage force division algorithm to find the coverage quality in WSNs. However, their algorithm can neither detect the coverage holes nor find the shape and size of a hole. In [33], the authors propose a novel method for describing the coverage holes graphically. Their proposed graphical hole description method is divided into two phases, namely coverage hole detecting and coverage hole describing. In [34], the authors present a coverage hole healing algorithm taking the existing nodes of the WSN to cover the holes. Their algorithm resolves a full coverage while minimizing the overlapped area of the sensing disks and deploying a minimum number of nodes.

**Figure 1:**
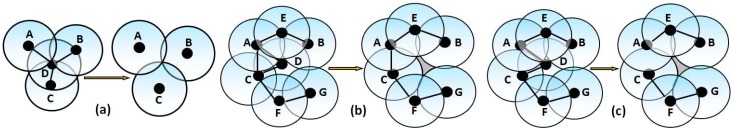
Example of the consequences of coverage holes. (**a**) Loss of connectivity due to death of node *D*; (**b**) Coverage hole due to death of node *D*; (**c**) Both coverage and connectivity hole due to death of node *D*.

It is to be noted that wireless sensors are deployed to monitor certain regions remotely for a wide range of potential applications, such as environment monitoring, object tracking, habitants monitoring and traffic control. If one or more sensors are dead in the network, the whole purpose of deploying sensors over the monitoring area becomes useless. As shown in Figure 1a, if the sensor *D* is dead, no communication is established among the sensors. As shown in Figure 1b, if sensor *D* is dead, coverage hole is created in the network, and the target within that area remains undetected. As shown in Figure 1c, if the sensor *D* is dead, both coverage, as well as communication holes are created, and therefore, the purpose of deploying sensors to detect any target remains futile.

In this paper, we propose a perimeter-based approach to find the coverage holes considering the points of intersection of the sensing discs of the wireless sensors deployed randomly over a monitoring region. The main contribution of this work over the existing hole detection protocols can be summarized as follows:

- A distributed coverage hole detection (DCHD) protocol is proposed to detect the coverage holes without the help of the sink by simply calculating the points of intersection of a node with its one-hop neighbors and checking them as covered or not.
- Algorithms are designed to filter the points of intersection into covered and non-covered points and then use this filtering method to detect the coverage hole. Such a filtering method can be done dynamically in an autonomous manner by maintaining the network integrity.
- Our proposed algorithms can detect the nature and shape of the coverage holes, which can be highly useful and make it easier to redeploy the nodes.

## 3. System Model

**Figure 2:**
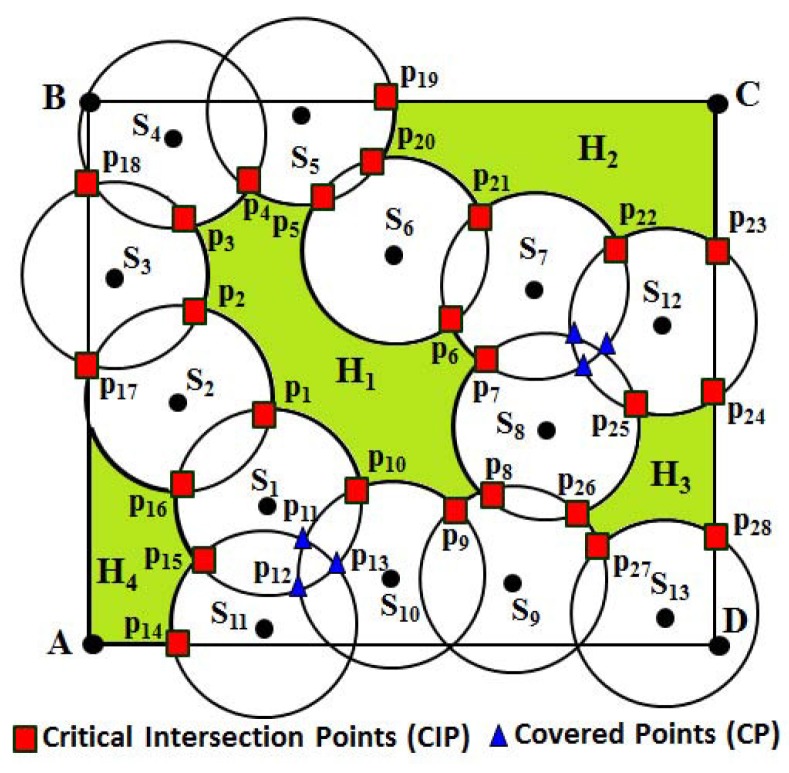
Example of coverage holes, critical intersection point (CIP) and covered point (CP).

Let us consider a wireless sensor network in which *n* number of wireless sensors are deployed randomly over a rectangular monitoring region *R*. Let $R_{c}$ and $R_{s}$ be the communication and sensing range of a sensor, respectively, such that $R_{c}=2R_{s}$. It is assumed that some part of the network is densely deployed with a large overlap of the sensing range, whereas some other part of the network is sparsely deployed, which is obvious due to the random deployment of the nodes. The part of the network in which nodes are deployed densely can have coverage overlapping, and the part of the network where nodes are sparsely deployed can have coverage holes; and the whole network is well connected irrespective of the presence of holes. Besides, coverage holes may be created due to the predictable death of the nodes, as some nodes may die due to power exhaustion. Similarly holes may be created due to unpredictable death of the nodes, as nodes may die due to hardware and software failures. Let $S=\{S_{1},S_{2},...,S_{i},...,S_{n}\}$ be the set of *n* nodes, where $S_{i}=\left(X_{i},Y_{i}\right)$, *i* = 1,2,3,...,*n* is the location of each sensor. It is assumed that every node knows its location information through GPS or some location services, and the coordinates of the monitoring region *R* are also known. As shown in Figure 2, $A(x_{1},y_{1})$, $B(x_{2},y_{2})$, $C(x_{3},y_{3})$ and $D(x_{4},y_{4})$ are the coordinates of the monitoring region *R*.

### Definitions

In this subsection, we define a few terms, which are used throughout the paper to develop our coverage hole detection protocol.

**Definition 1 (sensing disc):** The sensing disc of a sensor is the circle with the sensing range as its radius, when it is centered at its own location. Any object present within the sensing disc of a sensor is perfectly detected by it. Throughout the paper, the radius of the sensing disc is refereed to as its sensing range ($R_{s}$).

**Figure 3:**
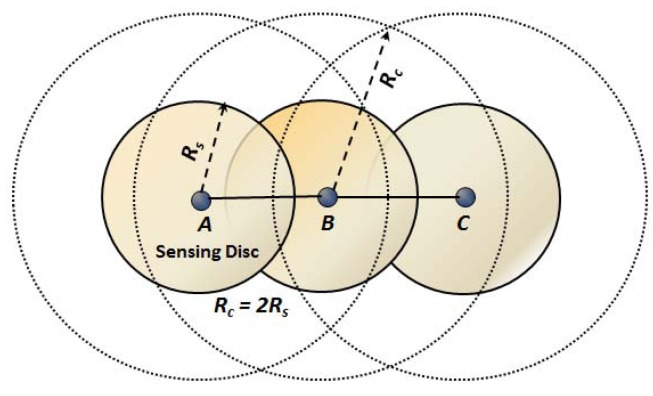
Example of the sensing disc and connecting neighbors.

**Definition 2 (connecting neighbors):** Two nodes *A* and *B* are said to be connecting neighbors, if their Euclidean distance d(A,B)$\leq R_{c}$. As per our assumption, communication range $R_{c}=2R_{s}$, and therefore, nodes *A* and *B* are connecting neighbors, as shown in Figure 3. Similarly, nodes *B* and *C* are the connecting neighbors. From this definition, it is clear that a connecting neighbor is always a one-hop neighbor of a node.

**Definition 3 (sensing neighbors):** Two nodes *A* and *B* are said to be sensing neighbors, if their Euclidean distance d(A,B)$<2R_{s}$. As shown in Figure 1, nodes *A* and *B* are sensing neighbors as d(A,B)$<2R_{s}$. Similarly, nodes *B* are *C* sensing neighbors as d(B,C) is $<2R_{s}$. In order to relate the sensing neighbors with connecting neighbors in our definition, it is clear that sensing discs of the sensing neighbors always overlap with each other, and they must be connecting neighbors, as they are located within one-hop from each other. As shown in Figure 3, nodes *A* and *B* are the sensing neighbors, as well as connecting neighbors. It is to be noted that throughout the paper, the set of sensing neighbors of any sensor $S_{i}$ is denoted as $N_{i}$.

**Definitions 4 (bounded/unbounded coverage hole):** If any part of the monitoring region is not covered by the sensing disc of any sensor, there exists a coverage hole. Coverage holes are found in a monitoring region, if some part of it is sparsely deployed. A coverage hole is said to be bounded, if it is enclosed by the sensing discs of the deployed sensors. Otherwise, it is an unbounded coverage hole. As shown in Figure 2, hole $H_{1}$ is a bounded coverage hole, whereas holes $H_{2}$–$H_{4}$ are unbounded coverage holes.

**Definitions 5 (boundary/non-boundary sensors):** Sensors that enclose a bounded coverage hole are called boundary sensors. If $S_{i}$ is the set of sensors ∀ *i*=1,2,3,...,*m*, for m≤n, and encloses a coverage hole; then, each element of the set of sensors $S_{i}$ is a boundary sensor. The sensors that enclose an unbounded coverage hole are called non-boundary sensors. As shown in Figure 2, sensors $S_{1}$ ∼$S_{10}$ are boundary sensors, as the coverage hole $H_{1}$ is enclosed by them. Similarly, sensors $S_{5}$, $S_{6}$, $S_{7}$ and $S_{12}$ are non-boundary sensors, as the coverage hole $H_{2}$ is enclosed by them. It is to be noted that a sensor could be a boundary, as well as a non-boundary sensor based on the presence of the holes. For example, as shown in Figure 2, sensors $S_{5}$, $S_{6}$ and $S_{7}$ are boundary, as well as non-boundary sensors, as they enclose the bounded coverage hole $H_{1}$ and unbounded coverage hole $H_{2}$, respectively.

**Definitions 6 (border/non-border sensors):** The sensor $S_{i}$, ∀*i*=1,2,3,...,*n* is said to be a border sensor, if its sensing disc intersects the border of the monitoring region. Otherwise, the sensor must be a non-border sensor. It is to be noted that the equation of each border line can be given as $y-y_{i}$= $\left(x-x_{i}\right)\left(\frac{y_{j}-y_{i}}{x_{j}-x_{i}}\right)$, ∀ i=1 and j=2 or i=2 and j=3 or i=3 and j=4 or i=1 and j=4. Similarly, ${\left(x-X_{i}\right)}^{2}+{\left(y-Y_{i}\right)}^{2}=R_{s}^{2}$ represents the equation of the sensing disc of any sensor, where $\left(X_{i},Y_{i}\right)$ is the location of a sensor *i*, ∀*i* = 1, 2, 3,..., *n*. As shown in Figure 2, $\bar{AB}$, $\bar{BC}$, $\bar{CD}$ and $\bar{DA}$ represent the equations of those borderlines.

**Definitions 7 (critical intersection points):** The critical intersection points (CIP) of a node are the points of intersection, which satisfy the conditions as follows. Let *I* be the set of points of the intersection of sensor $S_{i}$ with a sensing disc of its sensing neighbor $S_{j}$, for i≠j. The point $p_{ij}\in I$ is said to be a critical intersection point, if it is not covered by any other sensing neighbor $S_{k}$ of sensor $S_{i}$, for i≠k, or it lies on the border line of the monitoring region *R*. For example, as shown in Figure 2, the points in red color are the critical intersection points (CIP)due to the intersection of the sensing disc of one sensor with another or the sensing disc of one sensor with the border line of the monitoring region. It is to be noted that throughout the paper, the set of critical intersection points is denoted as *P*, where |P|≤|I|.

**Definitions 8 (covered points):** Let $S_{j}$ and $S_{k}$ be the sensing neighbors of a sensor $S_{i}$, for i≠j≠k. $p_{i}$ and $p_{j}$ are two points of intersection of the sensor $S_{i}$ with the sensing disc of its sensing neighbor $S_{j}$, such that both or any one of the points $p_{i}$ and $p_{j}$ are covered by the sensing disc of the sensor $S_{k}$. Then, the point(s) of intersection that is covered by the sensing disc of sensor $S_{k}$ is called the covered point (CP) of sensor $S_{i}$. For example, as shown in Figure 2, $S_{2}$, $S_{10}$ and $S_{11}$ are one-hop sensing neighbors of sensor $S_{1}$. There are several points of intersection of the sensing disc of $S_{1}$ with its sensing neighbors. However, only the red points $p_{1}$, $p_{10}$, $p_{15}$ and $p_{16}$ are the CIP of $S_{1}$, as they are not covered by any other sensors, whereas the blue points $p_{11}$, $p_{12}$ and $p_{13}$ are covered points (CP) of $S_{1}$, as shown in Figure 2, since they are covered by its sensing neighbors $S_{10}$, $S_{11}$ and itself. It is to be noted that a point of intersection of the sensing disc of one sensor with the sensing disc of another sensor could be either a *CIP* or a *CP*.

## 4. Proposed Hole Detection Protocol

In this section, a *distributed coverage hole detection* (DCHD) algorithm is designed to detect the bounded or non-bounded coverage holes present in the monitoring region. This scheme is proposed not only to solve the flaws of the perimeter-based coverage hole detection approaches, but also to consider the CIP to find a hole, which can reduce the time complexity of the coverage hole detection. In the first phase of the scheme, each sensor finds out its CIP set, as described in Section 4.1. In the second phase, each sensor also verifies if any of its points of intersection belong to a CP set or not, as described in Section 4.2. In the third phase, as described in Section 4.3, each sensor localizes and separates its CIP, which is used to determine the bounded or unbounded coverage hole. In the fourth phase, a sensor collaborates with its one-hop neighbors in the clock-wise direction and connect to its CIP to detect the presence of a coverage hole, as described in Section 4.4.

### 4.1. Determination of CIP

It is assumed that each sensor knows its own location information. After deployment of the nodes, each sensor exchanges its location information to know the location of its one-hop sensing neighbors. For simplicity, throughout the paper, sensing neighbors are refereed to as the neighbors. Upon receiving the location information from its one-hop neighbors, each sensor has to determine its CIP set (*P*) and *sensing neighbors set* (*N*). The algorithm for calculating the CIP is given in Algorithm 1. Upon executing the CIP calculation algorithm, each node has to maintain the list of its one-hop neighbor’s information and corresponding CIP, as given in Table 1. For example, the neighbors and CIP of different nodes given in Figure 2 can be shown in Table 1.

It is to be noted that there may be some isolated sensors deployed over the monitoring region *R*. Since the isolated sensors cannot have any sensing neighbors, they would not execute Algorithm 1, and there must be a coverage hole around that sensor. Suppose any sensor $S_{i}\notin$ isolate has to calculate its CIP. First, $S_{i}$ chooses its one neighbor $S_{j}$ from its neighbor set *N* and calculates the intersection points $p_{i},p_{j}$. If $p_{i}$ or $p_{j}$ is inside the monitoring region *R* and is not covered by any other neighbors of $S_{i}$, $p_{i}$ and $p_{j}$ become the elements of the CIP set *P*. Besides, $S_{j}$ is selected as the member of the sensing neighbors’ set *N*. This process continues for all of the neighbors of $S_{i}$.

For example, as shown in Figure 2, let us consider sensor $S_{1}$. The one-hop sensing neighbors’ set of $S_{1}$ can be $N\left(S_{1}\right)=\left\{S_{2},S_{10},S_{11}\right\}$. Since the sensing disc of $S_{1}$ intersects the sensing disc of $S_{2}$ and both points of intersection are not covered by any of its neighbor, points $p_{1}$ and $p_{16}$ becomes the CIP. Similarly, the sensing disc of $S_{1}$ also intersects the sensing disc of $S_{10}$, where the point of intersection $p_{12}$ is covered by another neighbor $S_{11}$, whereas the point $p_{10}$ is not covered by any of its neighbors. Hence, only point $p_{10}$ becomes the CIP. Thus, the CIP set of $S_{1}$ can be given as $P\left(S_{1}\right)=\left\{p_{1},p_{10},p_{15},p_{16}\right\}$. Based on Algorithm 1, after the sensor $S_{1}$ finds its CIP, its next hop sensing neighbor $S_{2}$ clockwise finds its one-hop sensing neighbors set, *i.e*., $N\left(S_{2}\right)=\left\{S_{3},S_{1}\right\}$, and its CIP set can be given as $P\left(S_{2}\right)=\left\{p_{1},p_{2},p_{16},p_{17}\right\}$. Thus, the process continues for all sensors, and finally, as shown in Figure 2, the set of CIP can be calculated as $P=\{p_{1},p_{2},...,p_{10},p_{14}∼p_{28}\}$.

**Algorithm 1** Algorithm for determining CIP of a node *i*.
1:**Input:**2:$N_{i}$: Set of all sensing neighbors of a node *i*;3:**Output:**4:$P_{i}$: Set of CIP of node *i*;5:**CIP Set Calculation Procedure:**6:Select any node *i* randomly;7:Initialize $P_{i}=\phi$;8:**if** ($N_{i}\neq \phi$)9:          {10:          Scan all members of $N_{i}$;11:          **if** (*i* is a non-border sensor)12:          {13:             Find intersection point *p* of node *i* with all members of $N_{i}$;14:             Find corresponding sensing neighbor *s* having an intersection point with node *i*;15:             Update $P_{i}=P_{i}⋃p$ and $N_{i}=N_{i}⋃s$;16:          }17:          **if** (*i* is a border sensor)18:          {19:             Scan all members of $N_{i}$;20:             Find point of intersection *b* of node *i* with border line of the monitoring region *R*;21:             Find point of intersection *p* of node *i* with all members of $N_{i}$;22:             Update $P_{i}=P_{i}⋃b⋃p$ and $N_{i}=N_{i}⋃s$;23:          }24:          }25:**else**26:          {27:             *i* is an isolated sensor;28:             Terminate the procedure;29:          }


### 4.2. Determination of CP

It is assumed that each node knows its *sensing neighbors set* (*N*). Taking the set of sensing neighbors set *N* as the input, each sensor can find out if the point of intersection with its one-hop neighbor is a CP or not. The details of the procedure for calculating a point as CP or not is given in Algorithm 2.

**Algorithm 2** Algorithm for determining the CP of a node *i*.
1:**Input:**$N_{i}$: Set of all sensing neighbors of node *i*;2:**Output:**$C_{i}$: Set of covered points of node *i*;3:**Calculation Procedure of CP set:**4:   Select any node *i* randomly;5:   Initialize $C_{i}=\phi$;6:   **if** ($N_{i}\neq \phi$)7:   {8:   Scan all members of $N_{i}$;9:   **if** (*i* is a non-border sensor)10:   {11:        Find intersection point of node *i* with *j*, which is one member of $N_{i}$;12:        Check if the point *p* is covered by a sensor *k*, which is another member of $N_{i}$ and j≠k;13:        **if** (*p* is covered by *k*)14:        *p* is a CP;15:        **else**16:        {17:          *p* is a CIP or a border point;18:          Execute Algorithm 1 to find CIP;19:        }20:   }21:   **else**22:   {23:        *i* is a border sensor;24:        Find point of intersection *b* of node *i* with border line of the monitoring region *R*;25:        Check if point *b* is covered by any neighbors of *i*;26:        **if** (point *b* is covered)27:        *b* is a CP;28:        **else**29:        Execute Algorithm 1 to find CIP;30:   }31:   }32:   **else**33:        *i* is an isolated sensor;


Let us consider an example, as shown in Figure 2. As shown in the figure, $N\left(S_{1}\right)=\left\{S_{2},S_{10},S_{11}\right\}$ is the one-hop sensing neighbors set of $S_{1}$. Based on Algorithm 2, let the sensor $S_{1}$ first find its one-hop neighbors and then find the point of intersection with its sensing neighbor $S_{10}$. $p_{10}$ and $p_{12}$ are the points of intersection of the sensing disk of $S_{1}$ with $S_{10}$, out of which $p_{12}$ is covered by its other neighbor $S_{11}$. Hence, $p_{12}$ becomes the CP, whereas $p_{10}$ becomes the CIP, as it is not covered by any other sensor of the network. Similarly, the sensing disc of the sensor $S_{11}$ can have points of intersection $p_{15}$ and $p_{13}$ with the sensing disc of the sensors $S_{1}$ and $p_{11}$ with the sensing disc of $S_{10}$. Here, points $p_{11}$ and $p_{13}$ are covered by the neighbors $S_{1}$ and $S_{10}$, respectively. Hence, the points of intersection $p_{11},p_{12}$ and $p_{13}$ become CP as marked in blue color in Figure 2. It is to be noted that a point of intersection that falls outside the monitoring region *R* is not taken into consideration for determining it as a CIP or CP.

### 4.3. Localization of CIP

**Figure 4:**
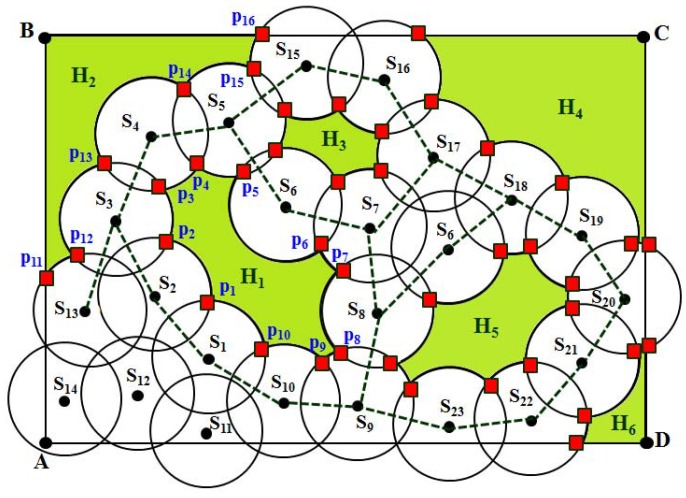
Example of the calculating of CIP and CP.

In this section, we describe how to localize all of the CIP to detect the coverage hole. As discussed in Section 4.1 and Section 4.2, the points of intersection of all of the sensors with their one-hop neighbors are classified into CP or CIP. However, a sensor may have more than one CIP at different sides of its sensing disc. It is to be noted that there is no confusion in detecting the coverage hole if the intersection point of a sensor has only one CIP. However, if a sensor has more than one CIP, it is necessary to localize them either on the lower or upper side of the line connecting the location of the sensor to the location of its neighboring sensor. For example, as shown in Figure 4, sensor $S_{3}$ has four different CIP, such as $p_{2},p_{3},p_{12}$ and $p_{13}$, out of which $p_{2}$ and $p_{3}$ are located at one side of the sensing disc of $S_{3}$, whereas $p_{12}$ and $p_{13}$ are located at the other side of its sensing disc. Then, it is important to know how to differentiate those points theoretically to detect the coverage holes remotely. In order to differentiate those points, first, connect the location of one sensor to the location of a one-hop neighbor of that sensor, as shown in Figure 4. For example, sensors $S_{2}$ and $S_{4}$ are the one-hop neighbors of $S_{3}$. Here, $p_{3}$ and $p_{13}$ are the CIP between the sensors $S_{3}$ and $S_{4}$. Now, draw a line $\bar{S_{3}S_{4}}$ between the centers of the sensing disc of sensors $S_{3}$ and $S_{4}$. Using simple coordinate geometry, it can be calculated that $p_{3}$ and $p_{13}$ lie below and above the $\bar{S_{3}S_{4}}$, respectively. Similarly, $S_{2}$ and $S_{13}$ are one-hop neighbors of $S_{3}$, and $p_{2}$ and $p_{12}$ are the CIP between the sensors $S_{2}$ and $S_{13}$, respectively. By joining the centers of the sensing disc of $S_{2}$ and $S_{3}$, it is observed that CIP $p_{2}$ lies below the line $\bar{S_{3}S_{2}}$, whereas CIP $p_{12}$ lies above the line $\bar{S_{3}S_{13}}$.

It is to be noted that the two points of intersection $p_{ij}$ and $p_{ji}$ between any two sensors $S_{i}$ and $S_{j}$ having locations $\left(x_{i},y_{i}\right)$ and $\left(x_{j},y_{j}\right)$, respectively, can be calculated as given in Equations (1) and (2), where $d_{ij}$ is the distance between sensors $S_{i}$ and $S_{j}$. Equation (3) represents the equation of the sensing disc of sensors $S_{i}$ and $S_{j}$. Let $p_{ij}$ = $\left(X_{1},Y_{1}\right)$ and $p_{ji}$ = $\left(X_{2},Y_{2}\right)$ be the two points of intersection between the sensors $S_{i}$ and $S_{j}$. Then,

(1) $$X_{1}:\frac{x_{i}+x_{j}}{2}+\frac{y_{j}-y_{i}}{2d_{ij}}\sqrt{\left(4R_{s}^{2}-d_{ij}^{2}\right)},Y_{1}:\frac{y_{i}+y_{j}}{2}-\frac{x_{j}-x_{i}}{2d_{ij}}\sqrt{\left(4R_{s}^{2}-d_{ij}^{2}\right)}$$

(2) $$X_{2}:\frac{x_{i}+x_{j}}{2}-\frac{y_{j}-y_{i}}{2d_{ij}}\sqrt{\left(4R_{s}^{2}-d_{ij}^{2}\right)},Y_{2}:\frac{y_{i}+y_{j}}{2}+\frac{x_{j}-x_{i}}{2d_{ij}}\sqrt{\left(4R_{s}^{2}-d_{ij}^{2}\right)}$$

(3) $$S_{i}:{\left(x-x_{i}\right)}^{2}+{\left(y-y_{i}\right)}^{2}=R_{s}^{2},S_{j}:{\left(x-x_{j}\right)}^{2}+{\left(y-y_{j}\right)}^{2}=R_{s}^{2}$$

Considering the location of each sensor and CIP, as given in the above equations, the procedure for localizing each CIP is given in Algorithm 3.

**Algorithm 3** Localization of CIP.
1:**Input:**2:   $N_{i}$: Set of all sensing neighbors of node $S_{i}$;3:   $S_{j}\in N_{i}$: Any sensing neighbor of node $S_{i}$;4:   $CIP_{ij}$ and $CIP_{ji}$: CIP between sensors $S_{i}$ and $S_{j}$;5:   $L_{ij}$: Set of all lines connecting to the location of sensor $S_{i}$ to location of $S_{j}$;6:**Output:**7:   $CIP_{L}$: Set of CIP below the line $L_{ij}$;8:   $CIP_{U}$: Set of CIP above the line $L_{ij}$;9:**Localization of CIP**10:   Initialize $CIP_{L}=\phi$ and $CIP_{U}=\phi$;11:   **Do**: for all CIP between sensors $S_{i}$ and $S_{j}$;12:   {13:          Select any sensor $S_{i}$;14:          Select another sensor $S_{j}$ from the set $N_{i}$;15:          Check location of $CIP_{ij}$ with respect to the line $L_{ij}$;16:   **if** ($CIP_{ij}$ is above $L_{ij}$)17:   {18:          Assign $CIP_{U}$←$CIP_{ij}$;19:          Update $CIP_{U}$;20:   }21:   **else**22:   {23:          Assign $CIP_{L}$←$CIP_{ij}$;24:          Update $CIP_{U}$;25:   }26:   }


### 4.4. Detection of Coverage Holes

In this section, the CIP are organized to detect the hole based on their location either above or below the line connecting to the location of any two neighboring sensors. The points that are below the connecting lines form a set of points as set $CIP_{L}$, whereas the points above the line form a set $CIP_{U}$. Then, in each set, those points are arranged in a clockwise fashion to check whether the nature of the hole is bounded or unbounded. By arranging the points either in the set $CIP_{L}$ or $CIP_{U}$ in the clockwise direction, a bounded coverage hole is detected if all elements of those sets form a loop, *i.e*., if the initial and terminal points are the same. However, an unbounded coverage hole is detected, if all elements of a set do not form a loop. It is to be noted that whether the coverage hole is bounded or unbounded is determined by the sensors that enclose them. The DCHD algorithm is run in each node to find the CIP and to detect the hole in collaboration with its one-hop neighbors. Once the hole detection procedure is terminated by the sensors, the information is transmitted to the sink by the sensor that has initiated this procedure. It could be possible that a node may be adjacent to more than one hole. In this case, the hole does not prevent the sensor from sending data to the sink. The node has to send information about the presence of both holes along with the nature of the holes to the sink. In our protocol, it is assumed that coverage holes are created due to the death of the nodes, and the role of the DCHD protocol is to detect the holes instead of detecting whether a node is dead or not. However, it is obvious that a node cannot have sensing overlapping with its one-hop neighbors, if a node is dead, and therefore, CIP is calculated and the hole detection done in the network.

**Figure 5:**
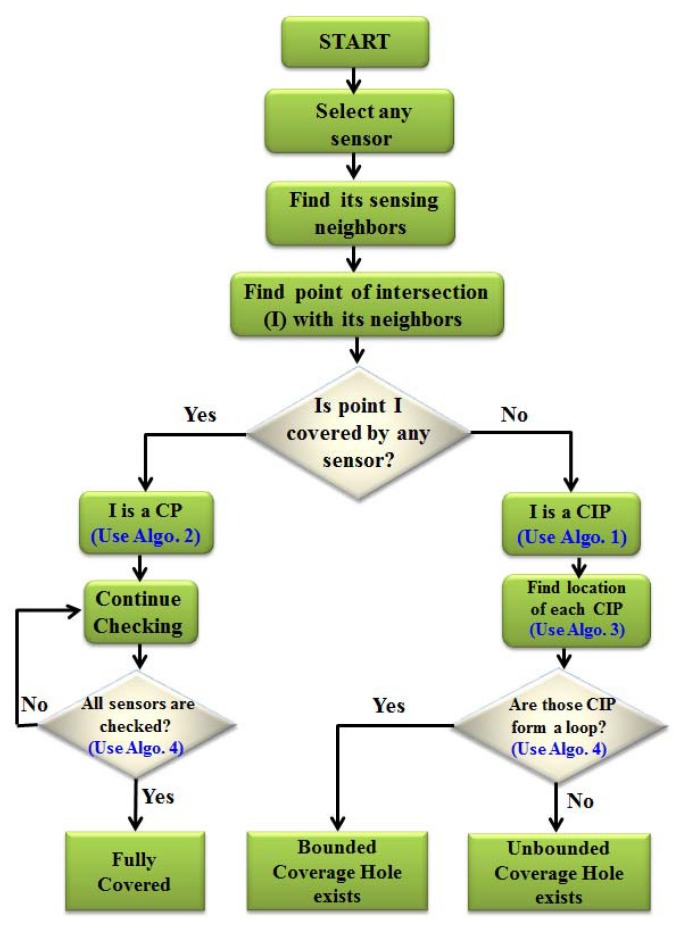
Flow chart of the complete coverage hole detection procedure.

In order to explain the detection of the coverage hole, let us take an example as shown in Figure 4. As discussed in the above subsection, the CIP are classified into two different sets based on their position. After calculating the position of each CIP either above or below the lines connecting to the centers of each sensing disc, two sets of points can be classified as sets $CIP_{L}$ and $CIP_{U}$. Thus, for the sensors $S_{1}$∼$S_{10}$, the set $CIP_{L}=\left\{p_{1},p_{2},p_{3},p_{4},p_{5},p_{6},p_{7},p_{8},p_{9},p_{10},p_{1}\right\}$, where $p_{1}$ is revisited, which implies that these points can form a loop, and therefore, a bounded coverage hole exists in between those sensors. Similarly, for the sensors $S_{2}$, $S_{13}$, $S_{3}$, $S_{4}$, $S_{5}$ and $S_{15}$, the set $CIP_{U}=\left\{p_{11},p_{12},p_{13},p_{14},p_{15},p_{16}\right\}$, where no point is revisited, which implies that though there is a coverage hole, the hole must be unbounded. It is to be noted that as shown in Figure 4, by connecting sensors $S_{17}$, $S_{18}$, $S_{6}$, $S_{8}$ and $S_{7}$, though those connecting lines can form a loop, no CIP is localized below those lines. This implies that all points of intersection between those sensors below the lines are detected as CP, and therefore, no coverage hole can be detected there. Taking all of the phases of the coverage hole detection procedure, the complete flow of the DCHD protocol can be shown as in Figure 5, and the DCHD algorithm is given in Algorithm. 4.

**Algorithm 4** Distributed coverage hole detection (DCHD) procedure.
1:**Input:**2:   $N_{i}$: Set of all sensing neighbors of node $S_{i}$;3:   $CIP_{L}$: Set of CIP below the connecting line;4:   $CIP_{U}$: Set of CIP above the connecting line;5:**Output:**6:   Type of coverage holes;7:**Detection of coverage holes:**8:   Select any sensor $S_{i}$;9:   Select another sensor $S_{j}$ from the set $N_{i}$;10:   Find points of intersection between $S_{i}$ and $S_{j}$; Let it be $P_{ij}$ and $P_{ji}$;11:   Select another sensor $S_{k}$ from the set $N_{i}$;12:   Check $P_{ij}$ and $P_{ji}$ are covered by $S_{k}$ or not;13:   **if** ($P_{ij}$ and $P_{ji}$ are not covered by $S_{k}$)14:   {15:        Assign $CIP_{ij}$←$P_{ij}$, $CIP_{ji}$←$P_{ji}$;16:        Execute Algorithm 3;17:        Function call: Hole_Type();18:   }19:   **if** ($P_{ij}$ || $P_{ji}$ is a CIP) (ex: Let $P_{ij}$ be a CIP)20:   {21:        Assign $CIP_{ij}$←$P_{ij}$;22:        Execute Algorithm 3;23:        Function call: Hole_Type();24:   }25:   **if** (both $P_{ij}$ and $P_{ji}$ are covered)26:   {27:        Assign CP←$P_{ij}$ and $P_{ji}$;28:        The network is fully covered;29:        Terminate the hole detection procedure;30:   }31:**Hole_Type()**32:          Arrange all points of $CIP_{L}$ and $CIP_{U}$ in clock-wise;33:          **if** (initial point == terminal point)34:          Hole is bounded;35:          **Else:** Hole is unbounded;


### 4.5. Construction of Covered and Uncovered Arcs

**Figure 6:**
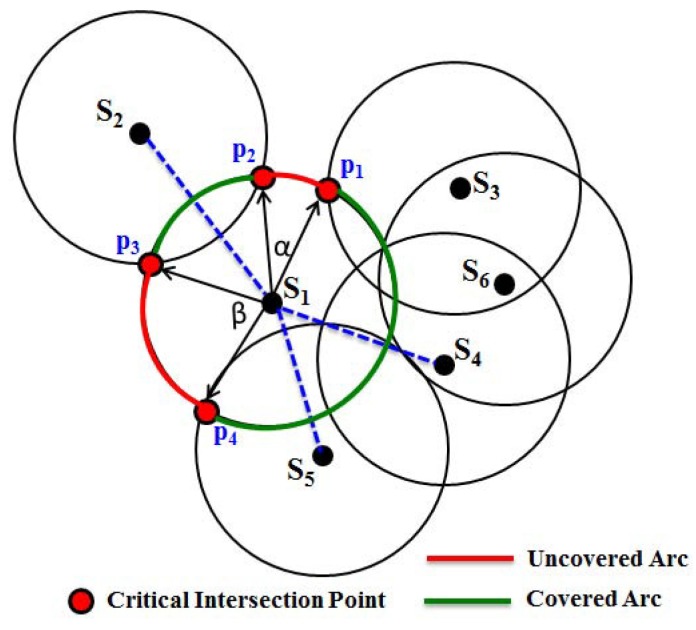
Construction of covered and uncovered arcs.

As discussed in the previous subsections, the presence of a coverage hole is detected by using the critical intersection points and after knowing their locations. However, it is essential to know the shape of the bounded or unbounded coverage holes. In this section, we propose a mechanism for how to get the shape of the hole by constructing covered and uncovered arcs. As shown in Figure 6, let $S_{2}$, $S_{3}$, $S_{4}$, $S_{5}$ and $S_{6}$ be the sensing neighbors of the node $S_{1}$. Here, $p_{1}$, $p_{2}$, $p_{3}$ and $p_{4}$ are the CIP of the sensor $S_{1}$. As discussed in the above subsection, it can be determined that $p_{1}$ and $p_{2}$ are located at one side of its sensing disc, whereas $p_{3}$ and $p_{4}$ are located at the other side of the sensing disc. It is to be noted that the location of all CIP and the position of each sensor are known. Since the locations of the one-hop neighbors of a sensor are known, we can connect the location of one sensor to another. Thus, as shown in Figure 6, first, the lines $\bar{S_{1}S_{2}}$, $\bar{S_{1}S_{3}}$, ..., $\bar{S_{1}S_{6}}$ are drawn, and the arcs are constructed as follows.

**Figure 7:**
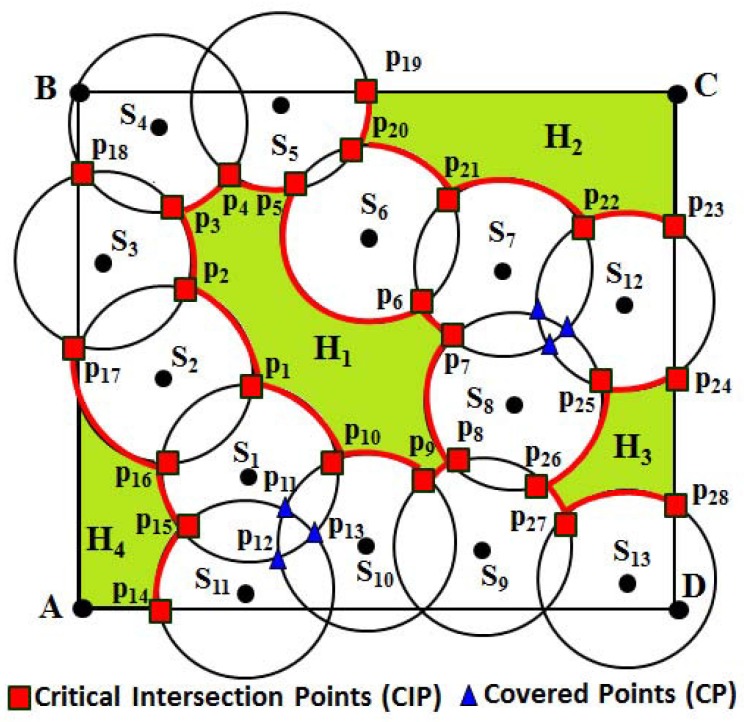
Formation of coverage holes using the uncovered arcs.

After locating the CIP, we have to construct the arcs along the circumference of the sensing disc of the sensor $S_{1}$. For this reason, first consider the points $p_{1}$, $p_{2}$ to draw the vectors $\vec{S_{1}p_{1}}$ and $\vec{S_{1}p_{2}}$ from the position of the sensor $S_{1}$. Let *α* be the angle between these two vectors, which can be calculated using the formula *α* = arccos$\left(\frac{\vec{S_{1}p_{1}}•\vec{S_{1}p_{2}}}{∥\vec{S_{1}p_{1}}∥∥\vec{S_{1}p_{2}}∥}\right)$. Then, the arc length of the arc can be calculated as $\hat{p_{1}p_{2}}$ = $\alpha \times R_{s}$. Thus, the arcs $\hat{p_{2}p_{3}}$, $\hat{p_{3}p_{4}}$ and $\hat{p_{4}p_{1}}$ are calculated. However, if any of those arcs intersect with the $\bar{S_{1}S_{2}}$, $\bar{S_{1}S_{3}}$, ..., $\bar{S_{1}S_{6}}$, they are considered as *covered arcs* and, therefore, are omitted for constructing the coverage hole. Based on this rule, as shown in the figure, only arcs $\hat{p_{1}p_{2}}$ and $\hat{p_{3}p_{4}}$ are considered as the *uncovered arcs* and are separated, as they do not intersect any of those connecting lines. Since arcs $\hat{p_{1}p_{2}}$ and $\hat{p_{3}p_{4}}$ are at different sides of the sensing disc, they are separated into two sets. Based on the initial point and terminal points of each arc, finally, they are connected with each other to construct the coverage hole by which, the shape of the hole can be known. For example, as shown in Figure 7, taking $S_{1}$ as the starting sensor, the arcs $\hat{p_{1}p_{2}}$, $\hat{p_{2}p_{3}}$, $\hat{p_{3}p_{4}}$, $\hat{p_{4}p_{5}}$, ∼$\hat{p_{10}p_{1}}$ marked in red are connected to each other, which can form a hole. Thus, these uncovered arcs enclose the bounded coverage hole $H_{1}$. Similarly, as shown in Figure 7, coverage holes $H_{2}$, $H_{3}$ and $H_{4}$ are enclosed by the arcs marked in red.

## 5. Performance Evaluation

In this section, the performance of our proposed DCHD protocol is evaluated in terms of control packet overhead, power consumption to detect the holes and time to detect the holes. Besides, our protocol is also compared to similar coverage hole detection protocols, such as the localized Voronoi polygon (LVP) [18] and the vector method [23]. The detailed description of the simulation setups and results is given as follows.

### 5.1. Simulation Setup

In our simulation, a rectangular monitoring region of a size of 500×400 m${}^{2}$ is considered. About 100 to 1500 sensor nodes are deployed randomly over the monitoring region, and the algorithms are simulated using ns-2.29 [35]. The sensing range of each sensor is taken to be 20 m, and communication range is fixed at 40 m, as we have considered that the communication range is twice the sensing range. The simulation parameters are setup according to the IEEE 802.15.4 MAC/PHY specification and the radio characteristics of IEEE 802.15.4 compliant product CC2420 [36] along with the AODV (*ad hoc* on-demand distance vector routing) protocol [37] and two-ray ground propagation model [38]. The initial energy of each node is assumed to have a fixed amount of 50 J of reserved energy, and the energy cost due to forwarding of each control packet is taken to be 0.3 J. Holes are generated randomly among the multi-hop and fully-connected nodes, so that they can form different groups of disjoint sets of nodes. Location information is given to all nodes, so that they can know about their one-hop sensing neighbors’ set and can find the point of intersection with the sensing disc of their neighbors. The data rate is kept as 250 Kbps, and the control packets are sent every 2 s to detect the neighbors, which is continued till 20 s to get the final list of one-hop neighbors of each node.

### 5.2. Simulation Result

**Figure 8:**
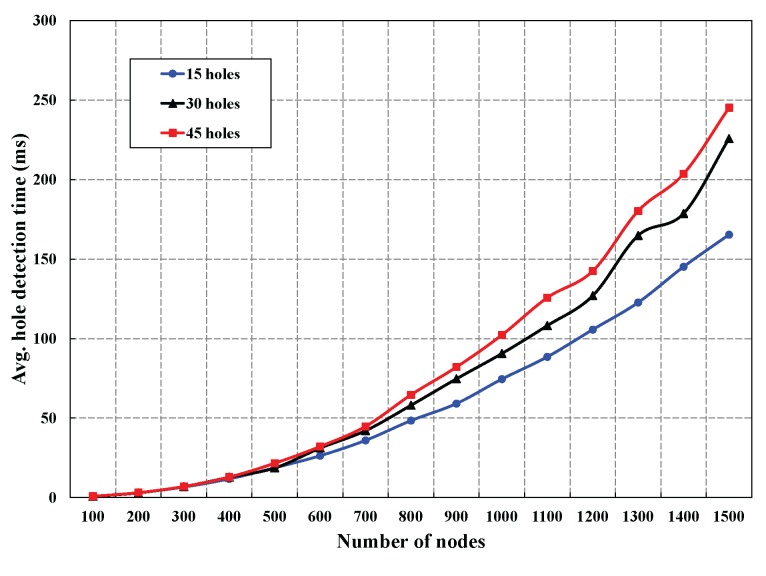
Average hole detection time in DCHD with different numbers of coverage holes.

In this section, first, we simulate our protocol for different numbers of coverage holes to evaluate the hole detection time, energy consumption and control packet overhead to detect the holes. Besides, we have also simulated our protocol to compare to other similar protocols in terms of control packet overhead, hole detection time and energy consumption with different numbers of holes and sensors. As shown in Figure 8, we have simulated the average hole detection time for different numbers of deployed sensors with the fixed number of coverage holes with 15, 30 and 45 present in the monitored region. It is observed that the average hole detection time is increased with the increase in the number of deployed sensors, which is quite reasonable. It is to be noted that the number of sensing neighbors of a node increases if the number of deployed nodes in the monitoring region is increased, which increases the hole detection time, as a node has to find its CIP and CP with its neighbors before detecting a hole.

**Figure 9:**
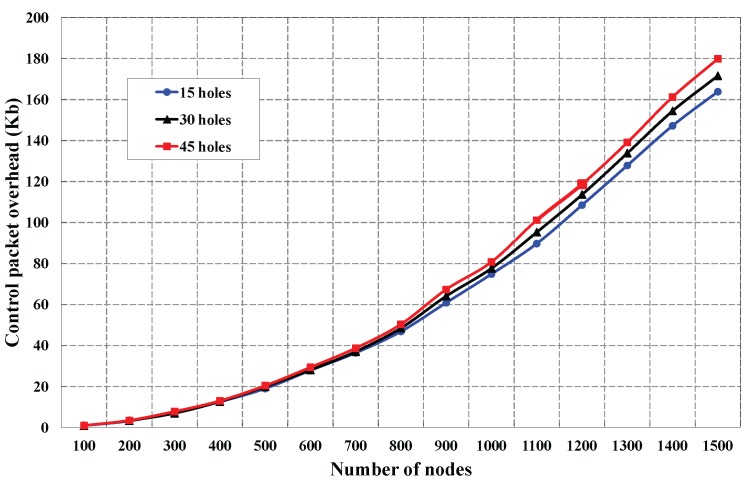
Control packet overhead in DCHD with different numbers of coverage holes.

**Figure 10:**
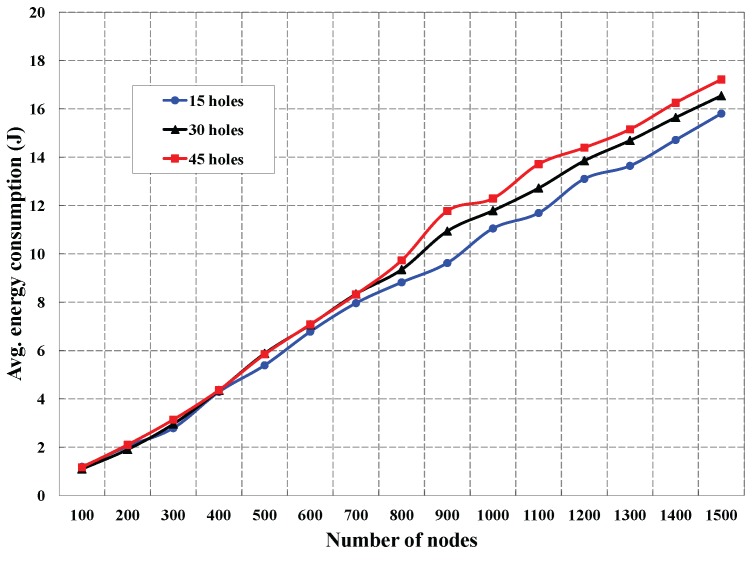
Energy consumption in DCHD to detect different numbers of coverage holes.

As shown in Figure 9, the control packet overhead is simulated with the number of deployed sensors. This simulation is carried out for different numbers of holes in the monitored region. We observe that the control packet overhead is increased, if the number of deployed sensors is increased. We find that the control packet overhead is almost similar for less deployed nodes (100 to 800 sensors) irrespective of the number of coverage holes, which is obvious due to the lesser density of the nodes around a coverage hole. However, control packet overhead shows a significant difference for different numbers of holes, when he number of deployed sensors is more than 800, as the node density increases around each hole. The analysis of the average energy consumption for different numbers of holes with different deployed sensors is presented in Figure 10. Let *k* be the number of neighbors of a sensor $S_{i}$ and $E_{t}$ and $E_{r}$ be the energy consumed by the sensor to transmit and receive data from its *k* neighbors. Since node $S_{i}$ has to broadcast its location information and has to receive location information from its *k* neighbors, the typical amount of energy consumption of a node in our DCHD protocol could be $Ec_{DCHD}$ = $E_{t}+kE_{r}$. As shown in Figure 10, it is observed that the average energy consumption is affected by different numbers of deployed sensors. This experiment is carried out for different number of holes, and it is found that the energy consumption is increased with the number of holes. This situation occurs as the degree of neighbors that enclose coverage holes increases due to the increase in the different numbers of holes.

**Figure 11:**
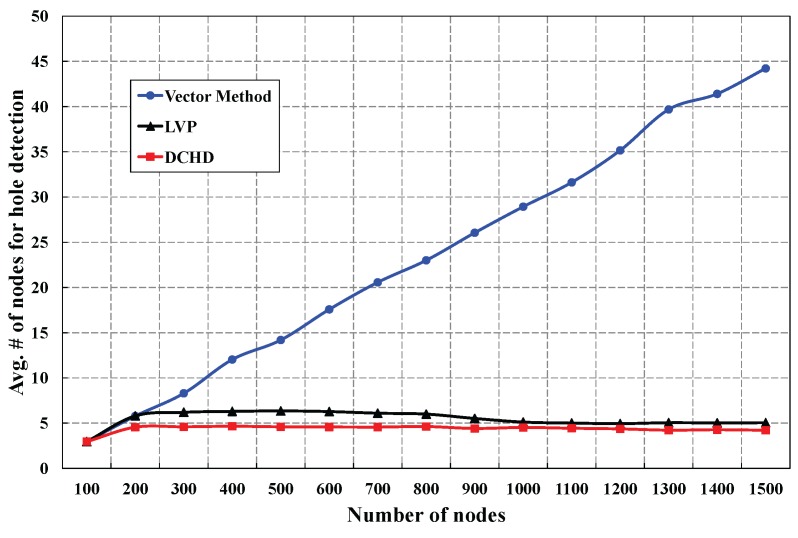
Comparison of DCHD in terms of the average number of nodes to detect a hole.

Figure 11 shows the average number of neighbors required to detect a coverage hole in LVP, the vector method and the DCHD algorithm, which is simulated with a fixed number of 15 holes in the monitoring region. When the number of sensors is 200, the average degree of neighbors is around 3.52, and therefore, the number of nodes required in hole detection is almost similar in all three protocols. However, when the number of sensor is increased to 800, the average degree of neighbors is around 27.31. Obviously, the average number of required nodes to detect the holes in the vector method is increased. However, in our DCHD and LVP algorithms, only a fixed number of nodes is used to detect the hole. However, in the DCHD algorithm, the average number of nodes needed for hole detection is 4.62, whereas an average of 6.02 nodes can participate in the LVP algorithm to detect the hole. From the simulation results depicted in Figure 11, we can infer that the execution time to find the hole in the vector method and the LVP algorithm is more than the DCHD algorithm. It is to be noted that the performance of DCHD is a little better than LVP in terms of the average number of nodes required for the hole detection. In LVP, the coverage hole is detected by taking the number of nearest neighbors, similarly to DCHD. According to their protocol, the communication range of each sensor is divided into four quadrants to find the number of nearest sensors of each sensor. In LVP, the number of neighbors that participate in the coverage hole detection procedure is a little more (6.02 nodes) than ours. Hence, the performance of DCHD over LVP is a little better in terms of the average number of nodes for the hole detection.

**Figure 12:**
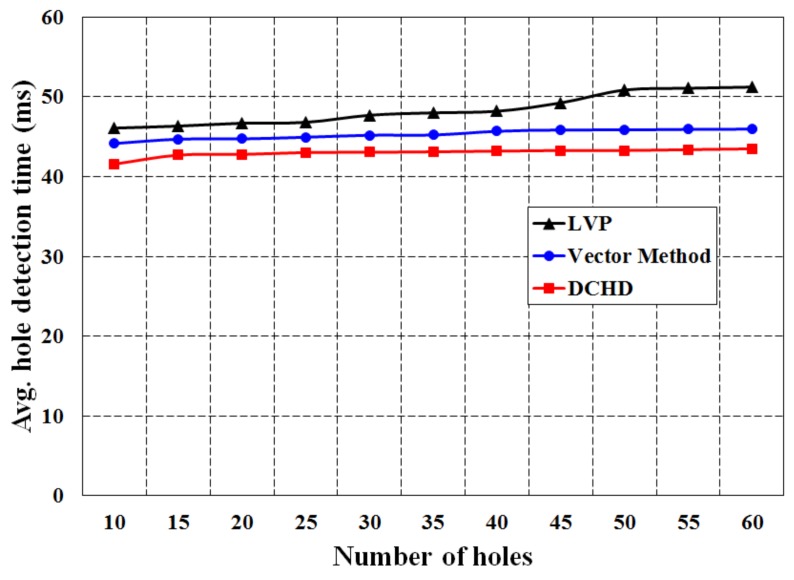
Comparison of DCHD with other protocols in terms of the average hole detection time.

**Figure 13:**
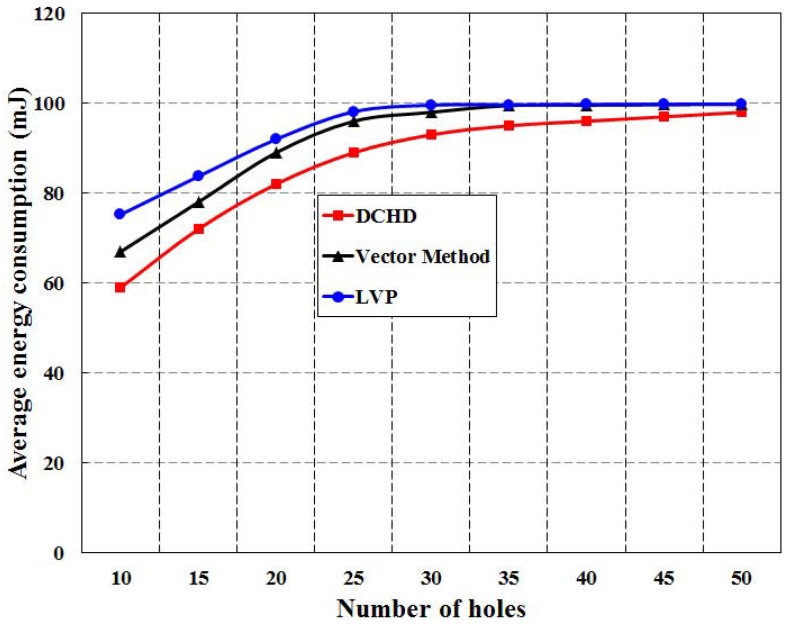
Comparison of DCHD with other protocols in terms of the average energy consumption.

Figure 12 shows the simulation result of average hole detection time with different numbers of holes for different protocols. It is noticed that the average hole detection time in DCHD is least as compared to LVP and the vector method. It is obvious from the results that DCHD outperforms LVP and the vector method, as fewer nodes in DCHD participate to detect the coverage holes. However, in all three protocols, the average hole detection time is increased with the increase in the number of holes, which is obvious. The average energy consumption in the three protocols is simulated for different numbers of coverage holes with a fixed number of nodes deployed over the monitoring region, which is fixed at 1000. As shown in Figure 13, it is found that the average energy consumption in these three protocols increases with the increase in the number of holes. However, the average energy consumption in DCHD is less than LVP and the vector method, which is due to the participation of less nodes to detect a coverage hole. Since, our protocol can save more power to detect the same number of holes as compared to other protocols, it can increase the network lifetime.

**Figure 14:**
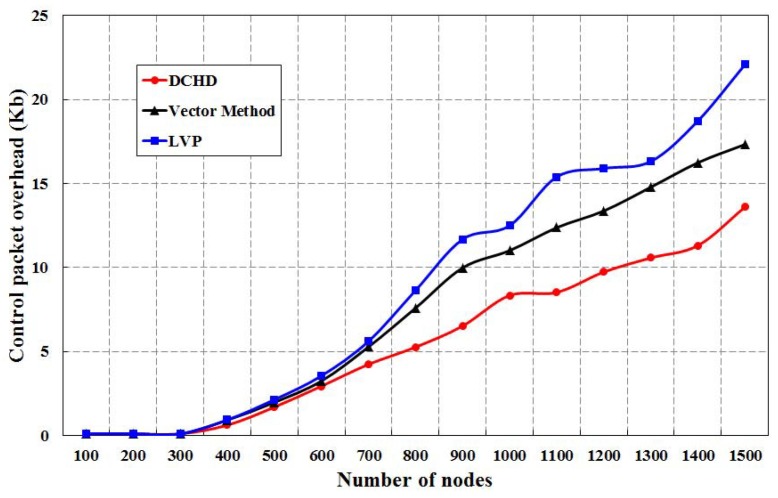
Control packet overhead of DCHD as compared to other protocols.

The control packet overhead of our protocol is compared to LVP and the vector method for different numbers of nodes, as shown in Figure 14. It is observed that our protocol outperforms LVP and the vector method when the number of coverage holes is less. From the simulation result, we find that there is no control packet overhead when the number of sensors is from 100 to 300. However, when the number of sensors is more than 300, the density of the sensors increases, and a greater number of redundant sensors in the network can increase the number of control packets.

## 6. Conclusion and Future Work

In this paper, a distributed coverage hole detection scheme is proposed in which the sensors can store location information of their one-hop neighbors to detect the presence of a coverage hole without the help of the sink. Prior to the detection of the holes, the construction of the holes is done using simple geometric methods to find the points of intersection and to check whether those points are covered or not. Algorithms are developed to join the critical points of intersection and, finally, to construct the hole. Using local information of the nodes, the global view of the coverage hole detection can be done. Besides, our protocol can formulate the bounded or unbounded coverage holes to predict the nature of the holes, which is a unique contribution in this work. Using covered or uncovered arcs, our protocol can also find the shape of the coverage hole. The interesting contribution in our work is that it can be applicable to detect the coverage holes irrespective of any shape or size of the monitoring region. Though we develop here the protocol for a rectangular monitoring region, our work with the same algorithm can be extended to the monitoring region of any shape and size, which is very practical in wireless sensor networks. Hence, our algorithms can be more useful and beneficial as compared to similar hole detection protocols. We feel that the proposed algorithm can find holes in the simplest way and with less time complexity and is therefore quite suitable for the memory and energy constraints of sensors. In our future work, we plan to design coverage hole detection algorithms for any irregular monitoring region. Besides, we will propose protocols for how to recover the coverage holes by moving a few sensors that have a large sensing overlap with their neighbors without disturbing the existing communication. Since the mobility of the sensors consumes more power, in our future work, we will design protocols to recover the holes with limited mobility.

## Figures and Tables

**Table 1:** List of neighbors and corresponding critical intersection points (CIP) of a node.

Node	Neighbors	Corresponding CIP
$S_{1}$	$S_{2}$	$P_{1}$, $P_{16}$
	$S_{10}$	$P_{10}$
	$S_{11}$	$P_{15}$
$S_{2}$	$S_{1}$	$P_{1}$, $P_{16}$
	$S_{3}$	$P_{2}$, $P_{17}$
$S_{3}$	$S_{2}$	$P_{2}$, $P_{17}$
	$S_{4}$	$P_{3}$, $P_{18}$
$S_{4}$	$S_{3}$	$P_{3}$, $P_{18}$
	$S_{5}$	$P_{4}$
$S_{5}$	$S_{4}$	$P_{4}$
	$S_{6}$	$P_{5}$, $P_{20}$
$S_{6}$	$S_{5}$	$P_{5}$, $P_{20}$
	$S_{7}$	$P_{6}$, $P_{21}$
$S_{7}$	$S_{6}$	$P_{6}$, $P_{21}$
	$S_{12}$	$P_{22}$
	$S_{8}$	$P_{7}$
$S_{8}$	$S_{7}$	$P_{7}$
	$S_{12}$	$P_{25}$
	$S_{9}$	$P_{8}$, $P_{26}$
$S_{9}$	$S_{8}$	$P_{8}$, $P_{26}$
	$S_{13}$	$P_{27}$
	$S_{10}$	$P_{9}$
$S_{10}$	$S_{1}$	$P_{10}$
	$S_{9}$	$P_{9}$
	$S_{11}$	No

## References

[1] Pottie G.J. Wireless sensor networks. Proceedings of the Information Theory Workshop.

[2] Pottie G.J., Kaiser W.J. (2000). Wireless integrated network sensors. Commun. ACM.

[3] Sohrabi K., Gao J., Ailawadhi V., Pottie G.J. (2000). Protocols for self-organization of a wireless sensor network. IEEE Pers. Commun..

[4] Heinzelman W.B., Murphy A.L., Carvalho H.S., Perillo M.A. (2004). Middleware to support Sensor Network Application. IEEE Netw. J..

[5] Boudriga N., Hamd M., Iyengar S. (2011). Coverage Assessment and Target Tracking in 3D Domains. Sensors.

[6] Sahoo P.K., Liao W.-C. (2015). HORA: Distributed Hole Recovery Algorithms for Wireless Sensor Networks. IEEE Trans. Mob. Comput..

[7] Zhang H., Hou J.C. (2005). Maintaining Sensing Coverage and Connectivity in Large Sensor Networks. Ad Hoc Sens. Wirel. Netw..

[8] Tian D., Georganas N.D. (2005). Connectivity Maintenance and Coverage Preservation in Wireless Sensor Networks. Ad Hoc Netw..

[9] Funke S. Topological hole detection in wireless sensor networks and its applications. Proceedings of the 2005 Joint Workshop on Foundations of Mobile Computing.

[10] Ahmed N., Kanhere S.S., Jha S. (2005). The Hole Problem in Wireless Sensor Networks: A Survey. ACM SIGMOBILE Mobile Computing and Communications Review.

[11] Xu H., Zhu J., Wang B. (2015). On the Deployment of a Connected Sensor Network for Confident Information Coverage. Sensors.

[12] Kumar S., Lai T.H., Balogh J. (2008). On *k*-coverage in a mostly sleeping sensor network. Wirel. Netw..

[13] Zhang H., Hou J.C. On the critical total power for asymptotic *k*-connectivity in wireless networks. Proceedings of the IEEE INFOCOM 24th Annual Joint Conference of the IEEE Computer and Communications Societies.

[14] Mo W., Qiao D., Wang Z. Lifetime maximization of sensor networks under connectivity and *k*-coverage constraints. Proceedings of the IEEE DCOSS.

[15] Meguerdichian S., Koushanfar F., Potkonjak M., Srivastava M.B. Coverage problems in wireless *ad hoc* sensor networks. Proceedings of The 20th Annual Joint Conference of the IEEE Computer and Communications Societies.

[16] Liu B., Towsley D. On the coverage and detectability of large-scale wireless sensor networks. Proceedings of the Workshop of Modeling and Optimization in Mobile, *Ad Hoc* and Wireless Networks.

[17] Shakkottai S., Srikant R., Shroff N. Unreliable sensor grids: Coverage, connectivity and diameter. Proceedings of the IEEE Societies INFOCOM 2003 Twenty-Second Annual Joint Conference of the IEEE Computer and Communications.

[18] Zhang C., Zhang Y., Fang Y. (2009). Localized algorithms for coverage boundary detection in wireless sensor networks. Wirel. Netw..

[19] Huang C.F., Tseng Y.C. The coverage problem in a wireless sensor network. Proceedings of the 2nd ACM International Conference on Wireless Sensor Networks and Applications.

[20] Zhang H., Hou J. (2005). Maintaining sensing coverage and connectivity in large sensor networks. Wirel. Ad Hoc Sens. Netw..

[21] Wang X., Xing G., Zhang Y., Lu C., Pless R., Gill C. (2005). Integrated coverage and connectivity configuration for energy conservation in sensor networks. ACM Trans. Sens. Netw..

[22] Liu C.Y. (2009). Remote Hole Detection Algorithms for Wireless Sensor Network. Master Thesis.

[23] Sahoo P.K., Tsai J.-Z., Ke H.L. Vector method based coverage hole recovery in Wireless Sensor Networks. Proceedings of the IEEE International Conference on Communication Systems and Networks.

[24] Kukunuru N., RajyaLakshmi D., Damodaram A. Hybrid approach for detecting and healing the coverage-hole in Wireless Sensor Network. Proceedings of the 2014 International Conference on Signal Propagation and Computer Technology (ICSPCT).

[25] Zhang Y., Zhang X., Wang Z., Liu H. Virtual edge based coverage hole detection algorithm in wireless sensor networks. Proceedings of the 2013 IEEE Wireless Communications and Networking Conference (WCNC).

[26] Zhang Y., Zhang X., Fu W., Wang Z., Liu H. (2014). HDRE: Coverage hole detection with residual energy in wireless sensor networks. Commun. Netw..

[27] Yu X., Xu M., Cheng L., Hu N. A novel coverage holes detection and holes recovery algorithm in wireless sensor networks. Proceedings of the Control and Decision Conference (CCDC).

[28] Htun A.M., Maw M.S., Sasase I. Reduced complexity on mobile sensor deployment and coverage holehealing by using adaptive threshold distance in hybrid Wireless Sensor Networks, Personal, Indoor, and Mobile Radio Communication (PIMRC). Proceedings of the 2014 IEEE 25th Annual International Symposium on Capital Hilton.

[29] Chintakunta H., Krim H. (2014). Distributed Localization of Coverage Holes Using Topological Persistence. IEEE Trans. Signal Process..

[30] Misra S., Singh S., Khatua M. (2015). MIRACLE: Mobility Prediction Inside a Coverage Hole Using Stochastic Learning Weak Estimator. IEEE Trans. Cybern..

[31] Fan X., Zhang Z., Lin X., Wang H. Coverage hole elimination based on sensor intelligent redeployment in WSN. Proceedings of the 2014 IEEE 4th Annual International Conference on Cyber Technology in Automation, Control, and Intelligent Systems (CYBER).

[32] Qin N., Zheng X., Tian G. (2015). Trajectory-Based Coverage Assessment Approach for Universal Sensor Networks. Sensors.

[33] Li W. (2014). A Novel Graphic Coverage Hole Description in Wireless Sensor Networks. IEEE Commun. Lett..

[34] Aliouane L., Benchaiba M. HACH: Healing Algorithm of Coverage Hole in a Wireless Sensor Network. Proceedings of the 2014 Eighth International Conference on Next Generation Mobile Apps, Services and Technologies (NGMAST).

[35] Network Simulator. http://www.isi.edu/nsnam/dist/.

[36] Texas Instruments Chipcon Products. http://www.chipcon.com.

[37] Perkins C.E., Royer E.M. *Ad-hoc* On-Demand Distance Vector Routing. Proceedings of the 2nd IEEE Workshop on Mobile Computing Systems and Applications.

[38] Rappaport T.S. (2002). Wireless Communications: Principles and Practice.

